# Mendelian randomization with invalid instruments: effect estimation and bias detection through Egger regression

**DOI:** 10.1093/ije/dyv080

**Published:** 2015-06-06

**Authors:** Jack Bowden, George Davey Smith, Stephen Burgess

**Affiliations:** ^1^MRC Biostatistics Unit, Cambridge Institute of Public Health, Cambridge, UK, ^2^MRC Integrative Epidemiology Unit, University of Bristol, Bristol, UK and ^3^Department of Public Health and Primary Care, University of Cambridge, Cambridge, UK

**Keywords:** Mendelian randomization, invalid instruments, meta-analysis, pleiotropy, small study bias, MR-Egger test

## Abstract

**Background:** The number of Mendelian randomization analyses including large numbers of genetic variants is rapidly increasing. This is due to the proliferation of genome-wide association studies, and the desire to obtain more precise estimates of causal effects. However, some genetic variants may not be valid instrumental variables, in particular due to them having more than one proximal phenotypic correlate (pleiotropy).

**Methods:** We view Mendelian randomization with multiple instruments as a meta-analysis, and show that bias caused by pleiotropy can be regarded as analogous to small study bias. Causal estimates using each instrument can be displayed visually by a funnel plot to assess potential asymmetry. Egger regression, a tool to detect small study bias in meta-analysis, can be adapted to test for bias from pleiotropy, and the slope coefficient from Egger regression provides an estimate of the causal effect. Under the assumption that the association of each genetic variant with the exposure is independent of the pleiotropic effect of the variant (not via the exposure), Egger’s test gives a valid test of the null causal hypothesis and a consistent causal effect estimate even when all the genetic variants are invalid instrumental variables.

**Results:** We illustrate the use of this approach by re-analysing two published Mendelian randomization studies of the causal effect of height on lung function, and the causal effect of blood pressure on coronary artery disease risk. The conservative nature of this approach is illustrated with these examples.

**Conclusions:** An adaption of Egger regression (which we call MR-Egger) can detect some violations of the standard instrumental variable assumptions, and provide an effect estimate which is not subject to these violations. The approach provides a sensitivity analysis for the robustness of the findings from a Mendelian randomization investigation.

Key Messages

- Mendelian randomization analyses using multiple genetic variants can be viewed as a meta-analysis of the causal estimates from each variant.
- If the genetic variants have pleiotropic effects on the outcome, these causal estimates will be biased.
- Funnel plots offer a simple way to detect directional pleiotropy; that is, whether causal estimates from weaker variants tend to be skewed in one direction.
- Under a weaker set of assumptions than typically used in Mendelian randomization, an adaption of Egger regression (MR-Egger) can be used to detect and correct for the bias due to directional pleiotropy.

## Introduction

Mendelian randomization^1^ is becoming an established method for testing whether a modifiable exposure has a causal role in the aetiology of a disease.^2^^,^^3^ As the subject moves forward, ever more ambitious analyses are being attempted. In particular, due to the proliferation of genome-wide association studies, the number of Mendelian randomization analyses using a large number of genetic variants is rapidly increasing.^4^^,^^5^ If the variants in total explain a larger proportion of the variance in the exposure, this will lead to more precise estimates of causal effects, thus increasing the power for testing causal hypotheses.^6^^,^^7^ However, an enlarged set of genetic variants is more likely to contain invalid instrument variables (IVs), due to violations of the assumptions necessary for valid causal inference. The issue of horizontal pleiotropy—where a genetic variant affects the outcome via a different biological pathway from the exposure under investigation—is a particular concern.^1^^,^^3^^,^^8^ The inclusion of pleiotropic variants in a Mendelian randomization analysis can lead to biased causal effect estimates and increased type I error rates for testing the causal null hypothesis.^9^ If the instrumental variable assumptions are violated, the findings of a Mendelian randomization analysis are open to the same criticisms as those levelled at traditional observational epidemiological analyses.^10^

In this paper, we view Mendelian randomization of a single study with multiple IVs as analogous to a meta-analysis. The overall causal estimate based on all the IVs can be interpreted as a weighted average of the individual IV estimates, just like a meta-analysis of separate study results. We show that bias resulting from pleiotropy is analogous to small study bias in meta-analysis,^11^ where small studies (with less precise estimates) tend to report larger estimates than big studies (with more precise estimates). One reason for this is that estimates from small studies with null findings tend not to be published. This induces a negative correlation across studies between the magnitude and precision of estimates. Publication bias is one aspect of the wider issue of dissemination bias.^12^ Moreover, there may be a host of complex, context-specific reasons that lead to differences between results from small and large studies in a specific meta-analysis. The general phenomenon is therefore prudently referred to under the umbrella term of ‘small study’ bias.^11^^,^^13^^,^^14^ In the context of Mendelian randomization with multiple instruments, we equate the precision of a single study’s estimate with the strength of a single instrument. Under certain assumptions, applying the regression method underlying Egger’s test—a method for assessing small study bias in meta-analysis^11^^,^^13–15^—is shown to give a consistent causal effect estimate even when all the genetic variants violate the standard IV assumptions.

In this paper, we describe a general statistical model for Mendelian randomization data with multiple potentially invalid instruments. Using the graphical representations of a scatter plot and a funnel plot, we discuss why the standard method of estimation, two-stage least squares (TSLS), may be biased when pleiotropy is present and when Egger regression can provide a consistent estimate of the causal effect. We apply both methods to data available from two published Mendelian randomization studies, and explore their performance further using simulated data. Finally, we emphasize that the method advanced here can strengthen or weaken evidence for a causal effect but, as for any single Mendelian randomization method, is itself subject to assumptions and limitations.

## Methods

We consider data from a Mendelian randomization study on *N* participants. For each participant, indexed by *i*, we measure *J* genetic variants ($G_{i1},G_{i2},\ldots ,G_{iJ}$), a modifiable exposure, ($X_{i}$) and an outcome ($Y_{i}$). We assume that confounders (represented by a single variable $U_{i}$) are unknown. The genetic variants are assumed to take the values 0, 1 or 2 (representing the number of exposure-increasing alleles of a bi-allelic single nucleotide polymorphism). The exposure is taken as a linear function of the genetic variants, the confounders and an independent error term ($\epsilon_{i}^{X}$). The coefficients $\gamma_{j}$ for each variant j represent the effects of the genetic variants on the exposure. The outcome is taken as a linear function of the genetic variants, the exposure, the confounders and an independent error term ($\epsilon_{i}^{Y}$). The causal effect of the exposure on the outcome is β. The coefficients $\alpha_{j}$ for each variant *j* represent the direct effects of the genetic variants on the outcome that are not mediated by the exposure. The total effect of each variant on the outcome comprises the direct effect ($\alpha_{j}$) and the indirect effect via the exposure ($\beta \gamma_{j}$).

(1) $$X_{i}=\sum_{j=1}^{J}\gamma_{j}G_{ij}+U_{i}+\epsilon_{i}^{X}$$

(2) $$Y_{i}=\sum_{j=1}^{J}\alpha_{j}G_{ij}+\beta X_{i}+U_{i}+\epsilon_{i}^{Y}.$$

Although the effects of the confounders on the exposure and on the outcome are taken as equal in equations (1) and (2), this assumption is not necessary and further parameters for these effects could be introduced into the model without affecting the methodological developments in this paper.

A genetic variant is a valid instrumental variable if the following assumptions hold:

- IV1: The genetic variant is independent of confounders *U*;
- IV2: The genetic variant is associated with the exposure *X*;
- IV3: The genetic variant is independent of the outcome *Y* conditional on the exposure *X* and confounders *U.*

**Figure 1.**
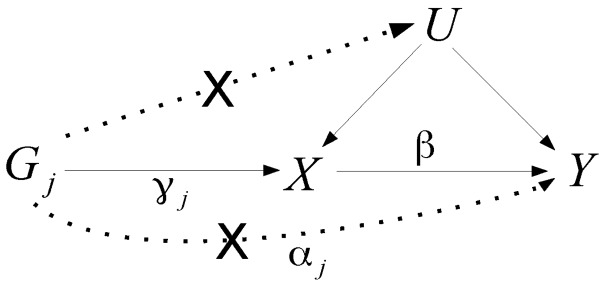
Illustrative diagram showing the standard instrumental variable assumptions for genetic variant $G_{j}$ (solid lines) with potential violations of the assumptions shown by dotted lines (which are marked with a ‘cross’). The genetic effect on the exposure X is $\gamma_{j}$, the direct genetic effect on the outcome Y is $\alpha_{j}$ and the causal effect of the exposure X on the outcome Y is β.

The second assumption implies that $\gamma_{j}\neq 0$ in equation (1). The third assumption is also referred to as ‘exclusion restriction’, and implies that $\alpha_{j}$ = 0 in equation (2); that is, an IV does not have an effect on the outcome when the exposure remains fixed.^16^ The IV assumptions and coefficients from equations (1) and (2) are represented in Figure 1

.

### Mendelian randomization and meta-analysis

With a single genetic variant *j*, the causal effect of the exposure on the outcome can be estimated using the ratio method (or Wald method^17^) as the coefficient from regression of the outcome on the genetic variant (denoted by ${\hat{\Gamma }}_{j}$) divided by the coefficient from regression of the exposure on the variant (denoted ${\hat{\gamma }}_{j}$).^18^ The reduced-form equation relating the outcome to the genetic variant *j* can be written as:

Yi=ΓjGij+ϵ′ijY=(αj+βγj)Gij+ϵ′ijY. 

If the genetic variant is a valid IV, $\alpha_{j}=0$ and the ratio method estimand (the quantity that is being estimated, denoted by $\beta_{j}$) is $\frac{\Gamma_{j}}{\gamma_{j}}=\frac{\beta \gamma_{j}}{\gamma_{j}}=\beta$.

With multiple genetic variants, the causal effect of the exposure on the outcome can be estimated using the TSLS method.^19^ The TSLS estimate is a weighted average of the ratio estimates calculated using each genetic variant in turn.^20^ If the genetic variants are uncorrelated (in linkage equilibrium), then the causal effect can be estimated from summarized data on the genetic associations with the exposure and with the outcome as:^21^

(3) $$\frac{\sum_{j=1}^{J}{\hat{\gamma }}_{j}^{2}\sigma_{Yj}^{-2}{\hat{\beta }}_{j}}{\sum_{j=1}^{J}{\hat{\gamma }}_{j}^{2}\sigma_{Yj}^{-2}}.$$

where ${\hat{\beta }}_{j}=\frac{{\hat{\Gamma }}_{j}}{{\hat{\gamma }}_{j}}$ is the ratio method estimate for variant *j*, and $\sigma_{Yj}$ is the standard error in the regression of the outcome on the *j*th genetic variant, assumed to be known. This same weighted average formula is used in a fixed-effect meta-analysis, where the IV-specific causal estimates ${\hat{\beta }}_{j}$ are the study-specific estimates, and the weights are the inverse-variance weights.^22^ This summarized estimate, which we refer to as an inverse-variance weighted (IVW) estimate, will differ slightly from the TSLS estimate in finite samples, as the correlation between independent genetic variants will not exactly equal zero,^23^ but the two estimates will be equal asymptotically (that is, they both tend towards the same quantity as their sample sizes increase towards infinity). However, an advantage of the IVW estimate is that it can be calculated from summarized data, whereas the TSLS estimate requires individual-level data. We assume for the remainder of the manuscript that the genetic variants are uncorrelated in their distributions (that is, knowledge of one does not help to predict the value of any other), as typically in Mendelian randomization one variant is taken from each gene region. Distantly located variants are usually uncorrelated; correlations between variants that are physically close can be found using an online tool such as [http://www.broadinstitute.org/mpg/snap/ldsearchpw.php].

If genetic variant *j* is not a valid IV, in particular because it has a direct effect on the outcome ($\alpha_{j}\neq 0$), then we have $\beta_{j}=\beta +\frac{\alpha_{j}}{\gamma_{j}}$ . The ratio estimate based on genetic variant *j* in an infinite sample will equal the true causal effect *β* plus an error term $\frac{\alpha_{j}}{\gamma_{j}}$ . In the same way, the TSLS and IVW estimates will tend towards:

$$\beta +\frac{\sum_{j=1}^{J}\gamma_{j}\sigma_{Yj}^{-2}\alpha_{j}}{\sum_{j=1}^{J}\gamma_{j}^{2}\sigma_{Yj}^{-2}}=\beta +\text{Bias}(\alpha ,\gamma ).$$

This implies that the TSLS estimate is consistent when the assumption IV3 is true and all the $\alpha_{j}$ parameters are zero. It is also consistent if the pleiotropic effects happen to cancel out, such that the bias term is equal to zero.^24^ Although this will not be universally plausible, we explore the condition that the correlation between the genetic associations with the exposure (the $\gamma_{j}$ parameters) and the direct effects of the genetic variants on the outcome (the $\alpha_{j}$ parameters) is zero. We refer to the condition that the distributions of these parameters are independent as InSIDE (Instrument Strength Independent of Direct Effect). It can be viewed as a weaker version of the exclusion restriction assumption. This relaxation of the IV assumptions was recently investigated by Kolesár *et al.*,^25^ although their work differs from ours and is not presented within the context of Mendelian randomization.

### Illustrative example

**Figure 2.**
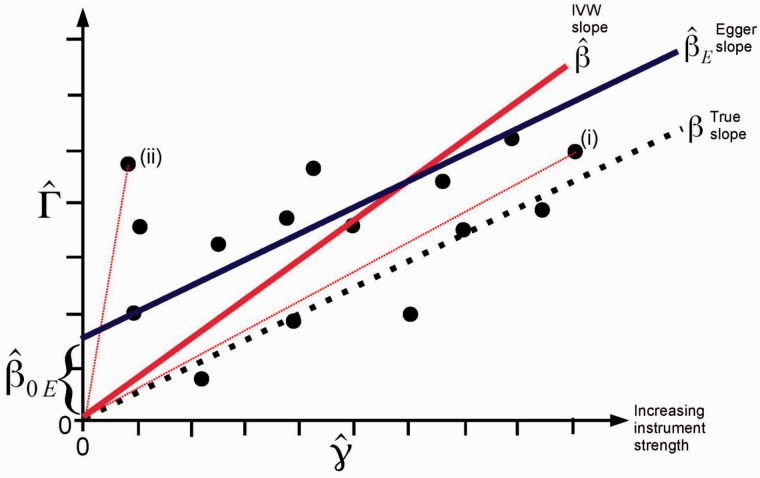
Plot of the gene–outcome ($\hat{\Gamma }$) vs gene–exposure ($\hat{\gamma }$) regression coefficients for a fictional Mendelian randomization analysis with 15 genetic variants. The true slope is shown by a dotted line, the inverse-variance weighted (IVW) estimate by a red line, and the MR-Egger regression estimate by a blue line. Refer to text for explanation of points (i) and (ii).

Illustrative data on the associations of multiple genetic variants with an exposure variable and with an outcome variable for 15 variants are displayed as a scatter plot in Figure 2. This is similar to a radial plot occasionally used in meta-analysis to display multiple estimates of the same quantity with a range of precisions.^26^ In this example, all of the IVs are invalid, but the InSIDE condition holds. The true causal effect is shown by the dotted line. The ratio estimates based on each genetic variant are the gradients of the slopes from the origin to the data point for that variant. The IVW estimate (shown by the solid red line) is the slope of the best fitting line through the data points that also passes through the origin. This is equal to the coefficient from a weighted regression of the gene–outcome association estimates (${\hat{\Gamma }}_{j}$) on the gene–exposure association estimates (${\hat{\gamma }}_{j}$) with the intercept constrained to zero, and weighted by the inverse of the precision of the IV–outcome coefficients ($\sigma_{Yj}^{-2}$).^27^ Here, all of the instruments are invalid, and so the slope of this line differs substantially from the true causal effect.

Under the InSIDE assumption, the numerator of the bias term of the ratio estimate for the *j*th genetic variant ${\hat{\alpha }}_{j}$ is independent of its denominator, ${\hat{\gamma }}_{j}$. This means that the bias of the ratio estimate ${\hat{\beta }}_{j}=\frac{{\hat{\Gamma }}_{j}}{{\hat{\gamma }}_{j}}$ is inversely proportional to $\gamma_{j}$. Consequently, ratio estimates for stronger genetic variants (ones with greater values of ${\hat{\gamma }}_{j}$), such as the variant marked (i) in Figure 2, will be on average closer to the true causal effect than those from weaker genetic variants, such as the variant marked (ii).

**Figure 3.**
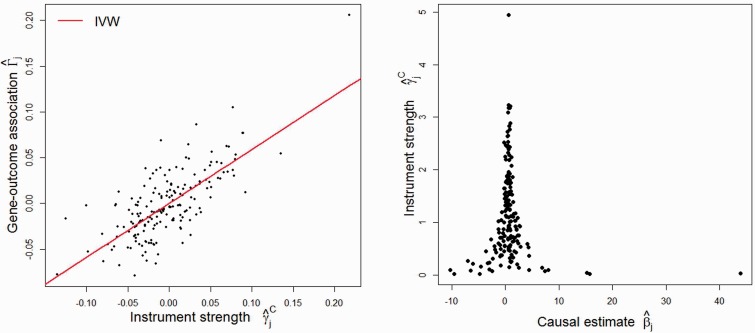
Genetic associations with height and lung function from 180 variants measured in the ALSPAC dataset. Left: scatter plot of genetic associations with forced vital capacity (${\hat{\Gamma }}_{j}$) against associations with height (${\hat{\gamma }}_{j}$), with causal estimate of height on lung function estimated by inverse-variance weighted method. Right: funnel plot of minor allele frequency corrected genetic associations with height (${\hat{\gamma }}_{j}^{C}$) against causal estimates based on each genetic variant individually (${\hat{\beta }}_{j}$).

**Figure 4.**
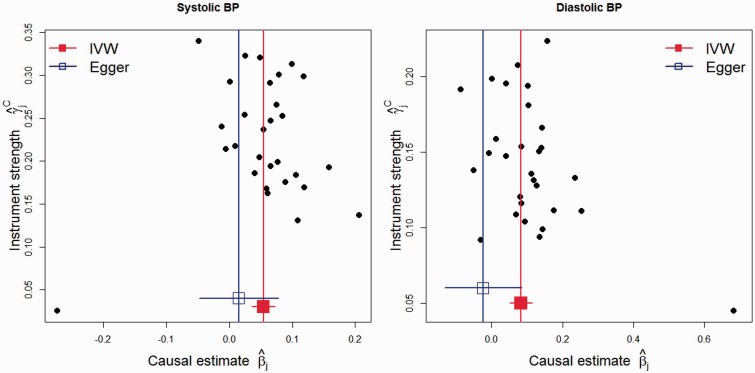
Genetic associations with blood pressure and coronary artery disease risk from 29 variants—funnel plots of minor allele frequency corrected genetic associations with blood pressure (${\hat{\gamma }}_{j}^{C}$) against causal estimates of blood pressure on CAD based on each genetic variant individually (${\hat{\beta }}_{j}$). Left: funnel plot for systolic blood pressure. Right: funnel plot for diastolic blood pressure. The inverse-variance weighted (IVW) and MR-Egger causal effect estimates are also shown.

The data can also be displayed visually by a funnel plot.^28^ In the context of meta-analysis, this is a plot of a measure of the precision of study estimates vs the estimates themselves (see Figures 3 and 4). Asymmetry in the funnel plot will occur if there is directional small study bias, as extreme results from smaller studies are more likely to be published. In the context of Mendelian randomization, we plot the genetic associations with the exposure ${\hat{\gamma }}_{j}$ against the individual IV estimates ${\hat{\beta }}_{j}$, as the genetic associations with the exposure are related to the precision of the IV estimates. We refer to asymmetry in this plot as ‘directional’ pleiotropy, meaning that the pleiotropic effects of genetic variants are not balanced about the null.

We consider regression of the ${\hat{\Gamma }}_{j}$ coefficients on the ${\hat{\gamma }}_{j}$ coefficients where the intercept is not constrained to be zero. We fit the linear model:

(4) $${\hat{\Gamma }}_{j}=\beta_{0E}+\beta_{E}{\hat{\gamma }}_{j}.$$

(We draw attention to the slight oddity in notation: the ${\hat{\Gamma }}_{j}$and ${\hat{\gamma }}_{j}$ association estimates are the data in this model, and *β*_0_*_E_* and *β_E_* are the coefficients in the regression model; estimates of these coefficients are also denoted by hats –${\hat{\beta }}_{0E}$ and ${\hat{\beta }}_{E}$.)

This model performs Egger regression, a special case of the general method of meta-regression.^11^ ^15^ Egger’s test for small study bias in meta-analysis assesses whether the intercept term *β*_0_*_E_* is different from zero. This will occur if the estimates from small studies (in the case of Mendelian randomization, estimates from weaker genetic variants) are more skewed towards either high or low values compared with estimates from large studies (stronger variants). The estimated value of the intercept in Egger regression ${\hat{\beta }}_{0E}$ can be interpreted as an estimate of the average pleiotropic effect across the genetic variants. An intercept term that differs from zero is indicative of overall directional pleiotropy.

It has been also asserted that ${\hat{\beta }}_{E}$ is a bias-reduced estimate for the true causal effect.^14^ Under model (1), we have the following equation for the slope coefficient from Egger regression:

$${\hat{\beta }}_{E}=\frac{\mathrm{cov}(\hat{\Gamma },\hat{\gamma })}{\mathrm{var}(\hat{\gamma })}=\hat{\beta }+\frac{\mathrm{cov}(\hat{\alpha },\hat{\gamma })}{\mathrm{var}(\hat{\gamma })}.$$

In the limit as both the sample size and the number of genetic variants increase to infinity, the InSIDE condition ensures that $\text{cov}(\hat{\alpha },\hat{\gamma })\overset{N\to \infty }{\to }\mathrm{cov}(\alpha ,\gamma )\overset{J\to \infty }{\to }0$ and therefore ${\hat{\beta }}_{E}$ is a consistent estimate of the causal effect *β.* This is illustrated by the solid blue line in Figure 2.

If genetic variants have different minor allele frequencies (MAFs), then a better measure of instrument strength can be constructed, as causal estimates from variants with low minor allele frequencies will have low precision. Provided that the genetic associations with the outcome are all estimated on the same individuals, the MAF-correction factors will be proportional to the standard errors of the gene–outcome associations $\sigma_{Yj}$ present in equation (3) under the assumption that the variant is in Hardy–Weinberg equilibrium, and so this correction is equivalent to performing Egger regression as a weighted linear regression using the $\sigma_{Yj}^{-2}$ as weights. The MAF-corrected weights are the same as those used by the IVW method in formula (3). If one uses the MAF-corrected weights within Egger regression, the InSIDE assumption must be that they are independent of the direct effects on the outcome. In order to distinguish our novel adaptation of Egger regression to Mendelian randomization from its original context, we will henceforth refer to its general application in this setting as `MR-Egger regression' and Egger's test as the ‘MR-Egger' test.

### Examples

To demonstrate this approach for the assessment of directional pleiotropy, we consider two illustrative examples of Mendelian randomization using many genetic variants that have been recently published. We use the available data on genetic associations with the exposure and with the outcome to construct a funnel plot and perform a visual inspection for asymmetry, as well as a formal statistical test using MR-Egger regression. We comment on the differences between the IVW causal effect estimate from equation (3), which assumes that all the genetic variants are valid IVs, and the MR-Egger estimate from equation (4), which makes assumptions IV1, IV2 and the InSIDE assumption.

### Causal effect of height on lung function

In a primarily methodological investigation of weak instrument bias, Davies *et al.*^29^ considered the causal effect of height (standardized) on lung function (measured as forced vital capacity, FVC, measured in ml) using 180 genetic variants as IVs with data on 3631 participants from the Avon Longitudinal Study of Parents and Children (ALSPAC) cohort.^30^ These variants were originally identified in a genome-wide association study.^31^ The associations of the variants with height and with FVC are displayed in a scatter plot in Figure 3 (left). The slope of the line through the scatter plot is the IVW causal effect estimate using all the variants as IVs of 0.59 [95% confidence interval (CI): 0.50, 0.67]. This is similar to the TSLS estimate of 0.60 (95% CI: 0.52, 0.68) reported by Davies *et al.* The causal estimates represent the increase in FVC for a 1 standard deviation increase in height.

Figure 3 (right) shows a funnel plot of the MAF-corrected genetic associations with the exposure against the individual causal effect estimates for each variant. A visual inspection of the funnel plot suggests that there is little asymmetry present. Applying MR-Egger regression with MAF-corrected weights to the summarized data yields an intercept estimate -0.0009 with an associated *P*-value of 0.75. The bias-adjusted causal effect estimate from MR-Egger regression is 0.60 (95% CI: 0.46, 0.75), a slight increase in magnitude and uncertainty compared with the IVW and TSLS estimates. There was also no apparent heterogeneity in the IV estimates from each genetic variant individually, as evidenced by Cochran’s *Q* test (*P* = 0*.*99). In the Web Appendix (available as Supplementary data at *IJE* online) we show how the IVW and MR-Egger regression methods were implemented on these data with just a single line of computer code (using R and Stata). In summary, there is no evidence that directional pleiotropy is an important factor for these data.

### Causal effect of blood pressure on coronary artery disease risk

Ehret *et al.*^32^ considered the causal effects of systolic blood pressure (SBP) and diastolic blood pressure (DBP; both measured in mmHg) on coronary artery disease (CAD) risk using 29 uncorrelated genetic variants. We consider data reported by Ehret *et al.* on genetic associations with blood pressure in over 200 000 individuals based on combined discovery and follow-up analysis, and data used by Ehret *et al.* from the CARDIoGRAM consortium on genetic associations with coronary artery disease in up to 22 233 cases and 64 762 controls of European descent (data available online at [www.cardiogramplusc4d.org]).^33^ The genetic associations with the exposure are corrected for varying minor allele frequencies; the MAF-corrected genetic associations are used in the figures and analyses.

Figure A1 displays scatter plots for SBP and DBP separately (available as Supplementary data at *IJE* online) and equivalent funnel plots are shown in Figure 4. A certain amount of asymmetry indicative of directional pleiotropy is present in them. For example, for SBP, 10 out of the 13 weakest variants have estimates greater than the IVW causal effect estimate.

For SBP, the IVW causal estimate of 0.054 (log odds ratio per 1 mmHg change in blood pressure) is far from the null (*P* = 4 × 10^−6^, odds ratio 1.055). In contrast, the MR-Egger test for the intercept gives a *P*-value of 0.21 and a causal estimate closer to the null (bias-corrected estimate: 0.015, *P* = 0*.*64, odds ratio 1.015). For DBP, the IVW causal estimate of 0.083 is again far from the null (*P* = 1 × 10^−5^, odds ratio 1.087). The MR-Egger test gives a *P*-value of 0.054 and a negative causal estimate (bias-corrected estimate: −0*.*024, *P* = 0*.*67, odds ratio 0.976). Additionally, Cochran’s *Q* test indicated strong evidence of heterogeneity between IV estimates based on the individual variants (*P < *0*.*001 for both SBP and DBP). Given the presence of apparent asymmetry in the funnel plots (Figure 4), the application of the MR-Egger test casts some doubt on the robustness of the original claims that these data allow generation of Mendelian randomization style analyses that provide strong support for the (well established) notion that blood pressure is causally related to coronary heart disease (CHD) risk.

Just as publication bias is not the only factor in a meta-analysis that would lead to an intercept estimate away from zero,^11^ ^13^ it does not necessarily imply that the genetic variants are pleiotropic in the Mendelian randomization context. Furthermore, it would be very surprising if blood pressure did not have some causal role in coronary artery disease (CAD) risk. Indeed, the wide confidence intervals for the causal effect of SBP and DBP on CAD risk obtained from MR-Egger regression are consistent with definitive analyses of the randomized trial evidence on the effectiveness of blood pressure-lowering treatments.^34^ It is therefore interesting to speculate what other mechanisms could be responsible for producing the asymmetry seen here. One alternative explanation is that the weaker genetic variants are more likely to be subject to the Beavis effect (also called ‘winner’s curse’).^35^ If genetic variants are chosen due to their association with the exposure in the dataset under analysis, then the association with the exposure is likely to be overestimated, and the association with the outcome could also then be overestimated due to confounding. This is known to lead to bias in Mendelian randomization estimates when there is overlap in the datasets used for estimating the genetic associations with the exposure and with the outcome (as is the case here).^36^ However, genetic associations with SBP and DBP from the replication analyses only were not reported in the original study, limiting the possibility to distinguish whether the asymmetry in the funnel plot is due to directional pleiotropy or the winner’s curse.

### Simulations

To further investigate the statistical properties of MR-Egger regression under realistic conditions, we perform a simulation study, generating artificial data with 25 genetic variants used as instrumental variables. We generate data in a two-sample Mendelian randomization setting, in which data on the genetic associations with the exposure and with the outcome are estimated in non-overlapping sets of individuals. Furthermore, we allow ourselves to make use of the summary data estimates only (e.g. the individual estimates for ${\hat{\gamma }}_{j},{\hat{\Gamma }}_{j},\text{ and }\sigma_{Yj}^{2},j=1,\ldots ,25$). The summarized data setting is increasingly common for applied Mendelian randomization investigations, such as the example of blood pressure and coronary artery disease risk. The IVW estimator was therefore felt to be the most natural implementation of the ‘standard’ approach to Mendelian randomization that could also be applied in this context, and so we chose this as our comparator. We expect its performance to closely mirror the two-sample two-stage least squares (TS2SLS) method,^37^ a variant of TSLS that can be applied to individual participant data in the two-sample setting, given their asymptotic equivalence. The simulations are repeated in the Web Appendix (available as Supplementary data at *IJE* online) in a one-sample setting. In this case, we found that the performance of standard one-sample TSLS and the IVW method were indeed highly similar.

We consider four scenarios for the pattern of pleiotropy, and consider the bias and coverage properties of the estimators with null and positive causal effects. The scenarios considered are:

- no pleiotropy, InSIDE assumption trivially satisfied (all the α parameters, representing the direct effects of genetic variants on the outcome, are equal to zero);
- balanced pleiotropy, InSIDE assumption satisfied (α parameters take positive and negative values);
- directional pleiotropy, InSIDE assumption satisfied (α parameters take only positive values, but are generated independently from the γ parameters, representing genetic effects on the exposure);
- directional pleiotropy, InSIDE assumption not satisfied (α parameters take positive values, and are correlated with the genetic effects on the exposure).

**Figure 5.**
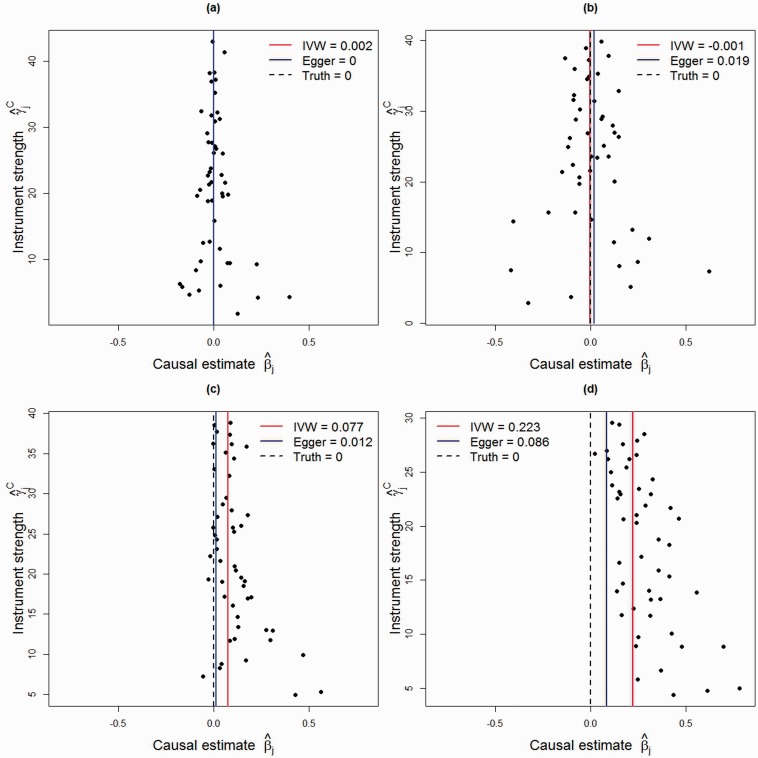
Funnel plots of minor allele frequency corrected genetic associations with exposure (${\hat{\gamma }}_{j}^{C}$) against causal estimates based on each genetic variant individually (${\hat{\beta }}_{j}$) for 50 IV estimates in four scenarios: (a) no pleiotropy; (b) balanced pleiotropy; (c) directional pleiotropy, InSIDE assumption satisfied; and (d) directional pleiotropy, InSIDE assumption not satisfied. The inverse-variance weighted (IVW, red) and MR-Egger (blue) causal effect estimates are also shown.

A possible situation corresponding to scenario (d) is that the pleiotropic effects of the genetic variants on the outcome act via a confounder. Specifically, if a genetic variant influences a confounder of the relationship between the exposure and outcome, then this will affect its associations with both the exposure and the outcome, leading to the InSIDE assumption being violated. Funnel plots illustrating data generated under each of these four scenarios for 50 genetic variants are provided in Figure 5. The details of parameters used in the simulation study and to produce Figure 5 are given in the Web Appendix (equivalent scatter plots for Figure 5 are also shown in Web Figure A2, available as Supplementary data at *IJE* online). We are particularly interested in the coverage properties of the estimators with a null causal effect, and the power of estimators with a positive causal effect, as salient findings from Mendelian randomization investigations are not only the magnitude of the causal effect—or indeed whether such can be estimated—but also whether a causal effect is present or absent.^1^^,^^38^

## Results

Results from the simulation study are given in Table 1 for 10 000 simulated datasets. Each row of the table corresponds to a particular simulation scenario and study size pairing. In each case, the mean F statistic across the 25 variants is also shown, in order to indicate the average instrument strength. This is a marker of the effective sample size of each scenario. The IVW and MR-Egger methods were implemented using weighted linear regression, as described in the Web Appendix (available as Supplementary data at *IJE* online). Standard errors and *P*-values (from t-tests) were taken directly from the regression output. All tests were two-sided and performed at a nominal significance level of 5%.

**Table 1.** Performance of inverse-variance weighted and MR-Egger regression estimates in simulation study for two-sample Mendelian randomization with a null (β = 0) and a positive (β = 0.05) causal effect. All tests are performed at 5% significance level

		Inverse-variance weighted		MR-Egger regression		
	Mean F	Mean estimate	Power to detect	Mean estimate	Power of	Power to detect
*N*	statistic	(mean SE)	causal effect	(mean SE)	MR-Egger test	causal effect
No causal effect: *β* = 0						
Scenario (a) no pleiotropy, InSIDE satisfied						
250	10.4	0.000 (0.022)	0.055	0.000 (0.047)	0.052	0.052
500	19.8	0.000 (0.015)	0.050	0.000 (0.035)	0.049	0.048
750	29.2	0.000 (0.013)	0.048	0.000 (0.030)	0.051	0.050
1000	38.6	0.000 (0.011)	0.049	0.000 (0.026)	0.048	0.050
Scenario (b) balanced pleiotropy, InSIDE satisfied						
250	10.4	0.000 (0.024)	0.052	0.001 (0.051)	0.047	0.050
500	19.8	0.000 (0.018)	0.052	0.000 (0.042)	0.051	0.050
750	29.2	0.000 (0.016)	0.048	0.000 (0.037)	0.053	0.049
1000	38.6	0.000 (0.015)	0.052	0.000 (0.034)	0.047	0.047
Scenario (c) directional pleiotropy, InSIDE satisfied						
250	10.4	0.034 (0.023)	0.293	0.010 (0.049)	0.089	0.055
500	19.9	0.036 (0.017)	0.530	0.005 (0.037)	0.142	0.055
750	29.2	0.036 (0.014)	0.689	0.004 (0.032)	0.195	0.054
1000	38.7	0.037 (0.013)	0.792	0.002 (0.029)	0.249	0.054
Scenario (d) directional pleiotropy, InSIDE violated						
250	10.6	0.115 (0.026)	0.989	0.037 (0.057)	0.330	0.110
500	20.2	0.119 (0.021)	1.000	0.026 (0.047)	0.545	0.099
750	29.7	0.120 (0.019)	1.000	0.022 (0.043)	0.651	0.088
1000	39.2	0.122 (0.018)	1.000	0.021 (0.041)	0.727	0.086
Positive causal effect: *β* = 0*.*05						
Scenario (a) no pleiotropy, InSIDE satisfied						
250	10.4	0.046 (0.022)	0.498	0.039 (0.049)	0.056	0.121
500	19.8	0.048 (0.016)	0.808	0.042 (0.037)	0.054	0.190
750	29.2	0.049 (0.013)	0.929	0.044 (0.031)	0.055	0.274
1000	38.6	0.049 (0.012)	0.977	0.045 (0.027)	0.053	0.347
Scenario (b) balanced pleiotropy, InSIDE satisfied						
250	10.4	0.046 (0.024)	0.439	0.039 (0.053)	0.051	0.109
500	19.8	0.048 (0.019)	0.675	0.042 (0.043)	0.054	0.155
750	29.2	0.049 (0.016)	0.810	0.044 (0.038)	0.055	0.199
1000	38.6	0.049 (0.015)	0.881	0.045 (0.035)	0.050	0.234
Scenario (c) directional pleiotropy, InSIDE satisfied						
250	10.4	0.080 (0.024)	0.890	0.048 (0.051)	0.110	0.148
500	19.9	0.084 (0.018)	0.995	0.047 (0.039)	0.174	0.215
750	29.2	0.085 (0.015)	1.000	0.048 (0.034)	0.227	0.276
1000	38.7	0.085 (0.013)	1.000	0.047 (0.030)	0.278	0.321
Scenario (d) directional pleiotropy, InSIDE violated						
250	10.6	0.161 (0.029)	1.000	0.074 (0.061)	0.359	0.226
500	20.2	0.167 (0.023)	1.000	0.066 (0.050)	0.562	0.261
750	29.7	0.169 (0.020)	1.000	0.065 (0.045)	0.661	0.290
1000	39.2	0.171 (0.019)	1.000	0.065 (0.042)	0.731	0.316

SE, standard error.

We first discuss results when there is a null causal effect of *β* = 0. In scenario (a) (no pleiotropy), both the IVW and MR-Egger regression return unbiased estimates for the causal effect, although mean standard errors from MR-Egger regression are generally twice as large as those from the IVW method. Rejection rates for the causal null hypothesis are controlled at the nominal 5% level for both methods. Additionally, rejection rates for the MR-Egger test are well controlled. The results for scenario (b) (balanced pleiotropy) are similar to (a) except that balanced pleiotropy has the effect of increasing the standard errors of the IVW and MR-Egger estimates by approximately 20% on average. Despite all instruments being invalid due to pleiotropy, rejection rates for the MR-Egger test of directional pleiotropy remain at 5%.

In scenario (c) (directional pleiotropy) the standard IVW estimate exhibits a marked bias. As the sample size increases, this bias becomes increasingly severe, and rejection rates for the causal null hypothesis increase from 30% to 80%. By contrast MR-Egger regression yields approximately unbiased estimates for *β* and type I error rates of the causal null hypothesis for the MR-Egger estimator remain around the 5% level. As the sample size increases, the power to detect directional pleiotropy rises modestly from 10% to just under 30%. In scenario (d) (directional pleiotropy), the InSIDE assumption does not hold. The pleiotropy due to a direct effect of variant *j* on the outcome ($\alpha_{j}$) is augmented with strong effect through a confounder of 2.5 times the magnitude of $\alpha_{j}$. This is a violation of causal assumption IV1, in addition to IV3. In this scenario, the standard IVW estimate exhibits such strong bias that the power to reject the causal null is essentially 1 for all sample sizes. MR-Egger regression is more robust to this strong violation of IV1, yielding estimates with a small amount of bias that decreases with increasing sample size. Likewise, rejection rates of the causal null hypothesis using MR-Egger regression are only slightly inflated. The power of the MR-Egger test to detect pleiotropy is also dramatically increased under scenario (d), being over 70% when *N* = 1000.

We now examine estimator performance with a positive causal effect of *β* = 0*.*05. In scenario (a) both methods exhibit a small amount of bias towards the null in their estimates for *β* for small sample sizes, with MR-Egger regression slightly more affected. This is in line with bias from weak instruments, which in a two-sample setting acts towards the null.^39^ As before, the IVW estimate is considerably more precise, and consequently has greater power to reject the causal null hypothesis. For the IVW approach, power increases from 50% to 98% as the sample size increases. For MR-Egger regression, power increases from only 12% to 35%. Although the power of the MR-Egger estimator to reject the causal null is low, error rates for the MR-Egger test of directional pleiotropy are still well controlled. The performance of both methods in scenario (b) is similar to (a), except the power to reject the causal null is reduced for both methods. In scenarios (c) and (d), the IVW estimate exhibits marked bias but very high power to reject the causal null, whereas MR-Egger regression yields approximately unbiased or minimally biased estimates and lower power.

In a second simulation we investigate the performance of the IVW method and MR-Egger regression under the causal null *β* = 0 in scenario (c), with a fixed sample size of *N* = 2000 but varying the number of genetic variants. The results are shown in Table 2. The bias of the IVW estimator reduces by just under 20% as the number of genetic variants *J* increases from 3 (very strong) instruments to 150 (weaker) instruments. However, this coincides with a reduction in the estimate’s standard error, so that its type I error rate rises sharply from 12% to 100%. MR-Egger regression returns approximately unbiased estimates for *β* for all values of *J.* As *J* increases the power of the MR-Egger test to detect directional pleiotropy increases from around 5% to 95%. The type I error rate of MR-Egger regression to detect a causal effect is well controlled for *J* ≤ 50 variants, but for over 100 variants some type I error inflation is apparent. In summary, MR-Egger regression works well with large numbers of genetic variants (in the sense that it has an increased power to detect pleiotropy), as long as the variants are not too weak.

**Table 2.** Performance of inverse-variance weighted and MR-Egger regression estimates ina simulation study for two-sample Mendelian randomization with a null causal effect (β* = 0*) and a fixed sample size, and varying the number of genetic variants (J)

		Inverse-variance weighted		MR-Egger regression		
*J*	Mean F statistic	Mean estimate (mean SE)	Power to detect causal effect	Mean estimate (mean SE)	Power of MR-Egger test	Power to detect causal effect
No causal effect: *β* = 0						
Scenario (c) directional pleiotropy, InSIDE satisfied						
3	407.0	0.042 (0.028)	0.127	0.003 (0.103)	0.059	0.054
5	295.0	0.039 (0.022)	0.248	0.000 (0.060)	0.085	0.050
10	172.0	0.038 (0.015)	0.580	0.001 (0.037)	0.166	0.051
15	121.0	0.037 (0.013)	0.780	0.000 (0.030)	0.248	0.048
20	93.6	0.037 (0.011)	0.894	0.001 (0.025)	0.329	0.055
30	64.4	0.037 (0.009)	0.980	0.001 (0.020)	0.475	0.052
50	39.8	0.036 (0.007)	1.000	0.002 (0.015)	0.667	0.058
100	20.7	0.035 (0.005)	1.000	0.005 (0.011)	0.877	0.082
150	14.2	0.035 (0.004)	1.000	0.007 (0.008)	0.944	0.150

## Discussion

In this paper we have proposed a simple sensitivity analysis for Mendelian randomization investigations using large numbers of genetic variants that may or may not have pleiotropic effects on the outcome of interest. Egger’s test is widely used as a tool for detecting small-study bias in meta-analysis. Under the InSIDE assumption that the direct pleiotropic effects of the genetic variants on the outcome are distributed independently of the genetic associations with the exposure, MR-Egger regression provides a valid test of directional (unbalanced) pleiotropy, and a valid test of the causal null hypothesis. Under this assumption, the slope estimate from MR-Egger regression is a consistent estimate of the true causal effect. When there are pleiotropic instruments but the InSIDE assumption is not satisfied, MR-Egger regression does not give a consistent estimate of causal effect, but remains a more robust method of inference compared with standard approaches which rely on the stronger assumption that there is no pleiotropy. This renders it an important sensitivity analysis tool in the Mendelian randomization context. In Box 1 we re-state the critical assumptions required for the valid application of MR-Egger regression and provide a step-by-step guide to its application in practice.

Box 1. Summary of assumptions for application of MR-Egger regression

- We take summarized genetic association estimates with the exposure (${\hat{\gamma }}_{1},\ldots ,{\hat{\gamma }}_{J}$), with the outcome (${\hat{\Gamma }}_{1},\ldots ,{\hat{\Gamma }}_{J}$), and standard errors of the genetic associations with the outcome ($\sigma_{Y1},\ldots ,\sigma_{YJ}$) for J genetic variants which are: (i) robustly associated with the exposure, (ii) uncorrelated with each other and (iii) in Hardy-Weinberg equilibrium. All variants must be orientated such that the genetic associations with the exposure have the same sign (that is, they must all be positive or all negative).
- For the standard inverse-variance weighted method, we perform a weighted linear regression of the genetic associations with the outcome on the genetic associations with the exposure, weighting by the inverse-variance of the genetic associations with the outcome ($\sigma_{Yj}^{-2}$). In this regression model, the intercept is constrained to equal zero. This analysis assumes that all genetic variants are valid instrumental variables.
- For the proposed MR-Egger method, we perform the same weighted linear regression with the intercept unconstrained. The intercept represents the average pleiotropic effect across the genetic variants (the average direct effect of a variant with the outcome). If the intercept differs from zero (the MR-Egger test), then there is evidence of directional pleiotropy. Under the assumption that the associations of the genetic variants with the exposure are independent of the direct effects of the genetic variants on the outcome (the InSIDE assumption), the slope coefficient from the MR-Egger regression is a consistent estimate of the causal effect. This is a weaker assumption than the assumption that all genetic variants are valid instrumental variables.The InSIDE assumption would be violated if the pleiotropic effects act via a confounder of the exposure—outcome association.
- R and Stata code to perform the inverse-variance weighted and MR-Egger methods is provided in the Web Appendix (available as Supplementary data at IJE online).

### Relation to existing literature

Several statistical methods have been proposed for consistent estimation of causal effects when the IV assumptions are not all satisfied. For example, Kang *et al.*^40^ propose a scenario in which only half of the genetic variants are required to be valid IVs. If infinite data were available, the identity of the valid IVs would be clear, as they would identify the same causal effect. Kang *et al.* provide an estimation method based on lasso penalization^41^ which not only gives consistent causal estimates in infinite samples, but also has reasonable finite sample properties. However, in contrast with the method proposed in this paper, which allows all the genetic variants to be invalid IVs, Kang *et al.* require at least half of the genetic variants to be valid IVs. Otherwise, if causal estimates from the two sets of valid and invalid genetic variants tended towards different values, it would not be possible to distinguish which of those values is the causal effect. A similar approach is simply to calculate the causal estimates using each genetic variant individually, rank the estimates in order of magnitude and take the median estimate.^42^ Again, this is guaranteed to give a consistent causal estimate if at least half of the genetic variants are valid IVs, although at the cost of a considerable reduction in precision of the causal estimate. Kolesár *et al.*^25^ also propose a consistent causal estimator under the same conditions as considered in this paper. This is based on a modified version of the bias-corrected TSLS estimator, which is part of the wider group of *k*-class estimators, a group that also includes the TSLS, bias-corrected TSLS and limited information maximum likelihood estimators.^43^ Further theoretical work is needed to compare the statistical properties of this estimator with the MR-Egger estimator proposed in this paper.

As Mendelian randomization with multiple IVs can be viewed as a meta-analysis of summarized genetic association estimates, methods and diagnostic tools developed for meta-analysis can also be used for Mendelian randomization. This is particularly relevant as summarized genetic association estimates from large consortia are increasingly becoming publicly available (such as those from the CARDIoGRAM consortium used in this paper).^44^ It has been shown that Mendelian randomization analyses based on summarized data are as efficient as those based on individual-level data.^23^ Other tools from the meta-analysis literature include methods for bias adjustment, such as the trim-and-fill method,^45^ and the use of pseudo-data.^46^ Another diagnostic tool is a heterogeneity test, which tests whether differences between estimates from different studies are compatible with chance variation.^47^ This can be performed using Cochran’s *Q* statistic.^44^ The null hypothesis is that the underlying association is the same in each study. In the Mendelian randomization context, we can test whether causal estimates from different genetic variants are compatible. Considerable heterogeneity would be evidence that the genetic variants are estimating different quantities, and would cast doubt on the IV assumptions being valid for all the variants. In IV analysis more generally, a heterogeneity test is equivalent to an over-identification test, often performed with individual-level data as part of a TSLS analysis.^48^

Another problem with the use of many genetic variants is that of weak instruments. With many IVs in a one-sample setting (genetic variants, exposure and outcome measured in the same participants), IV estimates (particularly those from the TSLS method) are biased in the direction of the observational association between the exposure and the outcome.^49^ This bias depends on the strength of the association of the IVs with the exposure, and is typically small if there is one IV^50^ or if the IVs are strongly associated with the exposure, but the bias may be substantial for Mendelian randomization in realistic settings.^36^ In a two-sample setting, weak instrument bias is in the direction of the null, and hence is a less serious problem, as it will not lead to false-positive findings.^37^^,^^39^ One solution proposed for weak instrument bias is the use of allele scores, whereby the number of exposure-increasing alleles across multiple genetic variants is summed across individuals.^9^ The total number of alleles (possibly weighted according to their association with the exposure) is then used as a single IV, rather than the genetic variants each being used as separate IVs. Provided that the weights are not taken from the data under analysis, this leads to estimates that are less affected by weak instrument bias. However, if results are solely given in terms of an allele score and not in terms of the individual variants, then inconsistency of causal estimates from different variants (either directional pleiotropy or heterogeneity) may not be evident. Failure of the MR-Egger test does not necessarily imply that the allele score estimate will be biased; however, it strongly suggests that bias may be an issue. It is therefore important not simply to report the associations of exposure and outcome with the allele score, but also associations with the genetic variants individually, such as in the scatter plot or funnel plot representations shown in this paper.

### Limitations of the proposed approach

Whereas the InSIDE assumption is plausible in some cases, it will not be valid in all circumstances, particularly if the pleiotropic effects of genetic variants act on confounders of the exposure–outcome association. This is because the confounders will induce a correlation between the direct effects of the variants on the outcome and the genetic associations with the exposure. This would occur, for example, in the case of population stratification. Another important way this could occur is if a genetic variant in truth affects an exposure causally upstream of the one under investigation (for example, if the exposure of interest is C-reactive protein but an included variant is associated with body mass index). However, in simulation scenario (d), where the pleiotropic effects through confounders (violating InSIDE) were 2.5 times larger than the direct pleiotropic effects (satisfying InSIDE), estimates from MR-Egger regression were much less biased and rejection rates of the causal null hypothesis were much closer to the nominal 5% rate than those from conventional IV methods. A related limitation is power—although the MR-Egger regression estimator was more robust, power to detect a causal effect was much reduced.

At present, we have not validated a universally reliable method for determining standard errors of the MR-Egger estimator when the causal effect is non-zero. Possible approaches include bootstrapping in a one-sample setting, or a hierarchical likelihood-based model that accounts for uncertainty in the genetic associations used as data points in the regression model. Other methods for giving valid inferences in the context of a meta-analysis and small-study bias have been discussed in the literature.^14^^,^^15^ The methods described in this paper therefore provide a sensitivity analysis to assess robustness of the conclusions of a Mendelian randomization investigation to potential bias from directional pleiotropy, and contribute to the overall evidence regarding the existence, direction and magnitude of the causal effect. If the MR-Egger estimate differs substantially from a conventional IV estimate, as in the example of blood pressure and coronary artery disease risk, the causal finding clearly requires additional interrogation. However, confidence intervals for the causal effect should be interpreted with caution when far from the null, for the reasons discussed.

In this article, we have assumed that genetic variants are uncorrelated. If the variants are correlated, and the correlations between variants are known, they can therefore be used within generalized weighted linear regression instead of weighted linear regression in either the IVW or MR-Egger method, incorporating the correlations into the weighting matrix. Further work is currently being undertaken to explore this method.

One further limitation of this approach is the assumption that the same causal effect is identified by multiple IVs. This assumption is not unique to our approach, as it is commonly made in IV analyses with multiple IVs. The presence of ‘treatment effect heterogeneity' is a complicating factor in causal analyses more generally, as it is not clear how to interpret a causal effect estimate if its magnitude depends on the nature of the intervention on the exposure. This is an important avenue for further research.

## Conclusion

In conclusion, the approaches of this paper should not be interpreted as a pretext for conducting Mendelian randomization analyses with large numbers of genetic variants without prior regard to the validity of the IV assumptions. However, they provide simple graphical and statistical methods that can detect some violations of the IV assumptions, and can therefore can used as a sensitivity analysis for assessing whether the effect estimation in a Mendelian randomization analysis is influenced by directional pleiotropic effects of the genetic variants.

## Supplementary Data

Supplementary data are available at *IJE* online.

## Funding

Jack Bowden is supported by an MRC Methodology Research Fellowship (grant MR/L012286/1). Stephen Burgess is supported by the Wellcome Trust (grant number 100114). George Davey Smith directs the MRC Integrative Epidemiology Unit supported by the MRC and the University of Bristol (MC_UU_12013_2, MC_UU_12013_5 and MC_UU_12013_8).

## Supplementary Material

Supplementary Data
