# Supplementary material for: Mendelian randomization with invalid instruments: effect estimation and bias detection through Egger regression
Source: Int J Epidemiol. 2015 Jun 6;44(2):512–25. doi: 10.1093/ije/dyv080 (PMC4469799; doi:10.1093/ije/dyv080)
Supplement: Supplementary Data [file supp_44_2_512__index.html]

Mendelian randomization with invalid instruments: effect estimation and bias detection through Egger regression — Supplementary Data 

# Mendelian randomization with invalid instruments: effect estimation and bias detection through Egger regression

## Supplementary Data

files

- Supplementary Data - docx file
