# Supplementary material for: Mendelian randomization with invalid instruments: effect estimation and bias detection through Egger regression
Source: Int J Epidemiol. 2015 Jun 6;44(2):512–25. doi: 10.1093/ije/dyv080 (PMC4469799; doi:10.1093/ije/dyv080)
Supplement: Supplementary Data [file supp_dyv080_IJEpaperFinal_updatedappendix_correctedJB.docx]

## Web Appendix

#### R code and results for the lung function example

# BetaYG    = vector of gene-outcome associations
# BetaXG    = vector of gene-exposure associations
# seBetaYG  = vector of gene-outcome association standard errors
# seBetaXG  = vector of gene-exposure association standard errors

# `>’ denotes the start of each block of R code 
 
#############################################
# IVW approach (with MAF corrected weights) #  
#############################################  

> IVWfit = summary(lm(BetaYG ~ -1+BetaXG,weights=1/seBetaYG^2))

> IVWfit$coef # Point estimate: note standard error is incorrect!
 
Coefficients:  
       Estimate Std. Error t value Pr(>|t|)  
BetaXG  0.58612    0.04387   13.36   <2e-16 ***  

# Inference with correct standard errors

> DF = length(BetaYG)-1

> IVWBeta = IVWfit$coef[1,1]

> SE = IVWfit$coef[1,2]/IVWfit$sigma

> IVW_p = 2*(1-pt(abs(IVWBeta/SE),DF))

> IVW_CI = IVWBeta + c(-1,1)*qt(df=DF, 0.975)*SE

# IVWResults = (point estimate, corrected standard error,

# 95% Confidence interval, t-statistic, p-value)

> IVWResults = c(IVWBeta,SE,IVW_CI,IVWBeta/SE,IVW_p)

####################################################  
# MR-Egger regression (with MAF corrected weights) #  
####################################################  
 
> BYG  = BetaYG*sign(BetaXG) # Pre-processing steps to ensure all  
> BXG  = abs(BetaXG)         # gene--exposure estimates are positive  
> MREggerFit = summary(lm(BYG ~ BXG,weights=1/seBetaYG^2))

> MREggerFit$coef # Point estimate: note standard errors are incorrect!

                 Estimate  Std. Error    t value     Pr(>|t|)  
(Intercept) -0.0008820533 0.002748935 -0.3208709 7.486846e-01  
BXG          0.6042841068 0.071675342  8.4308507 1.137390e-14

# Inference with correct standard errors

> MREggerBeta0 = MREggerFit$coef[1,1]

> MREggerBeta1 = MREggerFit$coef[2,1]

> SE0 = MREggerFit$coef[1,2]/MREggerFit$sigma

> SE1 = MREggerFit$coef[2,2]/MREggerFit$sigma

> DF = length(BetaYG)-2

> MRBeta0_p = 2*(1-pt(abs(MREggerBeta0/SE0),DF))

> MRBeta1_p = 2*(1-pt(abs(MREggerBeta1/SE1),DF))

> MRBeta0_CI = MREggerBeta0 + c(-1,1)*qt(df=DF, 0.975)*SE0

> MRBeta1_CI = MREggerBeta1 + c(-1,1)*qt(df=DF, 0.975)*SE1

# MREggerResults = (point estimate, corrected standard error,

# 95% Confidence interval, t-statistic, p-value) for

# intercept (row 1) and slope (row 2).

> MREggerResults = matrix(nrow = 2,ncol = 6)

> MREggerResults[1,] = c(MREggerBeta0,SE0,MRBeta0_CI,MREggerBeta0/SE0,MRBeta0_p)

> MREggerResults[2,] = c(MREggerBeta1,SE1,MRBeta1_CI,MREggerBeta1/SE1,MRBeta1_p)

# Bootstrap method to obtain confidence interval

# and standard error for slope parameter in

# MR-Egger regression (with MAF corrected weights)

> boot = NULL; straps = 10000

> for (i in 1:straps) {

BYG_boot = rnorm(length(BYG), mean=BYG, sd=seBetaYG)

BXG_boot = rnorm(length(BXG), mean=BXG, sd=seBetaXG)

BYG_boot = BYG_boot*sign(BXG_boot)   
 BXG_boot = abs(BXG_boot)

boot[i] = summary(lm(BYG_boot~BXG_boot,weights=seBetaYG^-2))$coef[2,1]

}

> boot_upper = sort(boot)[9751]

> boot_lower = sort(boot)[250]

> boot_se = sd(boot)

# MREggerBoot = c(point estimate, standard error, 95% confidence interval)

> MREggerBoot = c(MREggerBeta1,boot_se,boot_lower,boot_upper)

#### The standard error automatically reported for the inverse-variance weighted method is incorrect, as the residual standard error in a weighted regression analysis should be unity [Thompson and Sharp, Explaining heterogeneity in meta-analysis: a comparison of methods. Stat Med 1999; 18:2693-2708]. This can be corrected by dividing the reported standard error of the coefficient by the reported residual standard error [Burgess, Dudbridge, and Thompson, Re: “Multivariable Mendelian randomization: the use of pleiotropic genetic variants to estimate causal effects” (letter). Am J Epidemiol 2015; 181(4):290-291]. No correction to the coefficient is required.

#### The same correction to the standard error should be used in the MR-Egger method, and confidence intervals should be constructed using a t-distribution on the appropriate number of degrees of freedom (the number of genetic variants minus 2). Alternatively, a bootstrap approach is also recommended. The above code performs a parametric bootstrap by drawing association estimates from their estimated sampling distributions, and performing the MR-Egger method for each randomly drawn dataset. We show this for the slope parameter only. The authors would like to thank Jon White (University College London) for helpful discussions on writing the code for implementing the methods.

#### Stata code for performing the same analysis is given below.

#### Disclaimer: The R and Stata code given here is, to the best of our knowledge and at the time of publication, correct. Please contact the lead author if you have concerns.

#### Stata code

To implement the IVW approach (with MAF corrected weights) in Stata, the equivalent code is

regress BetaYG BetaXG [aw=1/seBetaYG^2], nocons

To implement MR-Egger regression (with MAF corrected weights) in Stata, the equivalent code is

regress BYG BXG [aw=1/seBetaYG^2]

where BYG and BXG have been derived from BetaXG and BetaYG as above.

The corrected standard error for the IVW method can be calculated by:

regress BetaYG BetaXG [aw=1/seBetaYG^2], nocons
local wrongse = _se[BetaXG]
local degfree = e(df_r)
predict BetaYG_fit
gen BetaYG_sqres = (BetaYG_fit-BetaYG)^2*seBetaYG^-2
summ BetaYG_sqres
local weighted_rss = r(sum)
di `wrongse'/sqrt(`weighted_rss'/`degfree')

#### The correction for the Egger method is similar.

#### Bootstrap confidence intervals for the MR-Egger method (with MAF corrected weights) can be calculated by:

gen BYG_boot = .
gen BXG_boot = .
set obs 10000
generate float boot = .

forvalues i = 1/10000 {
 quietly replace BYG_boot = rnormal(BetaYG, seBetaYG)
 quietly replace BXG_boot = rnormal(BetaXG, seBetaXG)
 quietly replace BYG_boot = BYG_boot * sign(BXG_boot)
 quietly replace BXG_boot = abs(BXG_boot)
 quietly regress BYG_boot BXG_boot [aw=1/seBetaYG^2]
 quietly replace boot = _b[BXG_boot] in `i'
 }

centile boot, centile (2.5 97.5)

#### Further details of the simulation study setup

In the simulation study, data were generated from the following model:

|  |  |
| --- | --- |
|  |  |
|  |  |

This is a more general version of model ([1](#x1-3001r1)) and ([2](#x1-3002r2)), in that it additionally allows genetic variant $G_{j}$ to be associated with confounding variable $U_{i}$. This occurs when is non-zero (a violation of IV1). If = 0 for all j, then this model reduces to equations ([1](#x1-3001r1)) and ([2](#x1-3002r2)). In all simulations: the $G_{ij}$ were generated from a trinomial distribution, taking values (0, 1, 2) with probabilities (0.49, 0.42, 0.09) – this is equivalent to a single nucleotide polymorphism with minor allele frequency 0.3; error variables were independently generated from a N(0, 2) distribution; and instrument strength parameters $\gamma_{j}$ were generated from a Uniform(0.5, 4) distribution.

The performance of the standard IVW method and MR-Egger regression were investigated in a two-sample Mendelian randomization analysis context with J = 25 variants, with a null (β = 0) and a positive (β = 0.05) causal effect. Two independent samples of N subjects were generated from the above model. For variant j out of 25, estimates for the gene-exposure associations ($\hat{\gamma}_{j}$) were obtained from the first sample and estimates for the gene-outcome associations ($\hat{\Gamma}_{j}$) were obtained from the second sample, in order to calculate the ratio estimates $\hat{\beta}_{j}=\hat{\Gamma}_{j}/\hat{\gamma}_{j}$. Simulation scenarios (a)–(d) were implemented by additionally specifying values for $\alpha_{j}$ and as below:

- No pleiotropy, InSIDE satisfied: $\alpha_{j}$ = 0, = 0;
- Balanced pleiotropy, InSIDE satisfied: $\alpha_{j}$ ~ Uniform(-0.2,0.2), = 0;
- Directional pleiotropy, InSIDE satisfied: $\alpha_{j}$ ~ Uniform(0,0.2), = 0;
- Directional pleiotropy, InSIDE not satisfied: $\alpha_{j}$ ~ Uniform(0,0.2), ~ Uniform(0,0.5).

In scenario (a), the ratio estimand based on the jth variant is equal to β. In scenario’s (b) and (c), the ratio estimand based on the jth variant is equal to $\beta+\frac{\alpha_{j}}{\gamma_{j}}$ but InSIDE holds. In scenario (d) the ratio estimand based on the jth variant is equal to

The InSIDE assumption is not satisfied in this case because the numerator of the bias term (which represents the total ‘direct’ effect not via the exposure) and its denominator (which represents the instrument strength) contain the common term ϕ_j_. Simulation results are shown in Table [1](#x1-90011).

Data for the four funnel plots shown in Figure [5](#x1-100015) were generated under the causal null hypothesis for scenarios (a)–(d), using the same two-sample approach. In order to accentuate the shapes of the funnel plots for illustrative purposes, we used J = 50 genetic variants and doubled the range of the Uniform sampling densities for $\alpha_{j}$ and . Web Figure [A2](#x1-220022) shows the equivalent scatter plots.

#### Results from the simulation study in a one-sample setting

The previous simulations were repeated in a one-sample Mendelian randomization setting. One sample of N subjects was generated, and estimates for the gene-exposure associations ($\hat{\gamma}_{j}$) and estimates for the gene-outcome associations ($\hat{\Gamma}_{j}$) were obtained from the same sample.

Results are shown in Table [A1](#x1-210011) and [A2](#x1-210022). The pattern of results is generally similar to the two-sample case, but both methods perform slightly worse in terms of small sample bias and Type I error rate inflation, and markedly worse with weak instruments. Weak instrument bias is more problematic in the one sample context because it acts in the direction of the confounded observational association. It appears to be slightly worse for estimates from MR-Egger regression, while Type I error rate is worse for estimates from the IVW method.

These simulations were repeated using standard one-sample TSLS instead of IVW as the comparator method, and the results were very similar (data not shown). We conclude that IV analysis with weak instruments in a one-sample setting is troublesome, and that these difficulties are not resolved by the application of MR-Egger regression.

|  |  |  |  |  |  |  |
| --- | --- | --- | --- | --- | --- | --- |
|  |  |  |  |  |  |  |
|  |  | Inverse-variance weighted | | MR-Egger regression | | |
|  |  |  |  |  |  |  |
|  | Mean F | Mean estimate | Power to detect | Mean estimate | Power of | Power to detect |
| N | statistic | (mean SE) | causal effect | (mean SE) | MR-Egger test | causal effect |
|  |  |  |  |  |  |  |
| No causal effect: β = 0 | | |  |  |  |  |
|  |  |  |  |  |  |  |
| Scenario (a) – no pleiotropy, InSIDE satisfied | | |  |  |  |  |
|  |  |  |  |  |  |  |
| 250 | 10.4 | 0.005 (0.021) | 0.077 | 0.016 (0.046) | 0.062 | 0.075 |
| 500 | 19.8 | 0.003 (0.015) | 0.067 | 0.011 (0.035) | 0.058 | 0.067 |
| 750 | 29.2 | 0.002 (0.013) | 0.060 | 0.009 (0.030) | 0.058 | 0.068 |
| 1000 | 38.6 | 0.002 (0.011) | 0.056 | 0.007 (0.026) | 0.056 | 0.060 |
|  |  |  |  |  |  |  |
| Scenario (b) – balanced pleiotropy, InSIDE satisfied | | |  |  |  |  |
|  |  |  |  |  |  |  |
| 250 | 10.4 | 0.005 (0.023) | 0.081 | 0.017 (0.051) | 0.058 | 0.075 |
| 500 | 19.9 | 0.003 (0.018) | 0.065 | 0.011 (0.041) | 0.056 | 0.068 |
| 750 | 29.2 | 0.002 (0.016) | 0.058 | 0.009 (0.037) | 0.053 | 0.063 |
| 1000 | 38.6 | 0.002 (0.014) | 0.063 | 0.007 (0.034) | 0.054 | 0.062 |
|  |  |  |  |  |  |  |
| Scenario (c) – directional pleiotropy, InSIDE satisfied | | |  |  |  |  |
|  |  |  |  |  |  |  |
| 250 | 10.4 | 0.043 (0.022) | 0.470 | 0.035 (0.047) | 0.052 | 0.124 |
| 500 | 19.8 | 0.040 (0.016) | 0.644 | 0.022 (0.037) | 0.081 | 0.100 |
| 750 | 29.2 | 0.039 (0.014) | 0.770 | 0.017 (0.032) | 0.113 | 0.086 |
| 1000 | 38.6 | 0.039 (0.012) | 0.853 | 0.013 (0.029) | 0.159 | 0.080 |
|  |  |  |  |  |  |  |
| Scenario (d) – directional pleiotropy, InSIDE violated | | |  |  |  |  |
|  |  |  |  |  |  |  |
| 250 | 10.6 | 0.128 (0.023) | 0.998 | 0.087 (0.052) | 0.129 | 0.386 |
| 500 | 20.2 | 0.126 (0.020) | 1.000 | 0.061 (0.045) | 0.326 | 0.280 |
| 750 | 29.7 | 0.126 (0.018) | 1.000 | 0.050 (0.042) | 0.467 | 0.232 |
| 1000 | 39.3 | 0.125 (0.017) | 1.000 | 0.043 (0.039) | 0.576 | 0.202 |
|  |  |  |  |  |  |  |
|  |  |  |  |  |  |  |
| Positive causal effect: β = 0.05 | | |  |  |  |  |
|  |  |  |  |  |  |  |
| Scenario (a) – no pleiotropy, InSIDE satisfied | | |  |  |  |  |
|  |  |  |  |  |  |  |
| 250 | 10.4 | 0.055 (0.021) | 0.678 | 0.067 (0.046) | 0.063 | 0.306 |
| 500 | 19.8 | 0.053 (0.015) | 0.886 | 0.061 (0.035) | 0.058 | 0.397 |
| 750 | 29.2 | 0.052 (0.013) | 0.962 | 0.059 (0.030) | 0.059 | 0.479 |
| 1000 | 38.6 | 0.052 (0.011) | 0.988 | 0.058 (0.026) | 0.056 | 0.560 |
|  |  |  |  |  |  |  |
| Scenario (b) – balanced pleiotropy, InSIDE satisfied | | |  |  |  |  |
|  |  |  |  |  |  |  |
| 250 | 10.4 | 0.056 (0.023) | 0.609 | 0.068 (0.051) | 0.059 | 0.277 |
| 500 | 19.9 | 0.053 (0.018) | 0.786 | 0.062 (0.041) | 0.057 | 0.317 |
| 750 | 29.2 | 0.052 (0.016) | 0.868 | 0.059 (0.037) | 0.053 | 0.345 |
| 1000 | 38.6 | 0.052 (0.014) | 0.911 | 0.058 (0.034) | 0.054 | 0.371 |
|  |  |  |  |  |  |  |
| Scenario (c) – directional pleiotropy, InSIDE satisfied | | |  |  |  |  |
|  |  |  |  |  |  |  |
| 250 | 10.4 | 0.093 (0.022) | 0.968 | 0.085 (0.047) | 0.051 | 0.426 |
| 500 | 19.8 | 0.090 (0.016) | 0.999 | 0.073 (0.037) | 0.080 | 0.483 |
| 750 | 29.2 | 0.089 (0.014) | 1.000 | 0.067 (0.032) | 0.112 | 0.523 |
| 1000 | 38.6 | 0.089 (0.012) | 1.000 | 0.063 (0.029) | 0.157 | 0.565 |
|  |  |  |  |  |  |  |
| Scenario (d) – directional pleiotropy, InSIDE violated | | |  |  |  |  |
|  |  |  |  |  |  |  |
| 250 | 10.6 | 0.179 (0.023) | 1.000 | 0.138 (0.052) | 0.127 | 0.710 |
| 500 | 20.2 | 0.176 (0.019) | 1.000 | 0.112 (0.045) | 0.322 | 0.661 |
| 750 | 29.7 | 0.176 (0.018) | 1.000 | 0.101 (0.042) | 0.463 | 0.642 |
| 1000 | 39.3 | 0.175 (0.017) | 1.000 | 0.093 (0.039) | 0.572 | 0.622 |
|  |  |  |  |  |  |  |
|  |  |  |  |  |  |  |

Web Table A1: Performance of inverse-variance weighted and MR-Egger regression estimates in simulation study for one-sample Mendelian randomization with a null (β = 0) and a positive (β = 0.05) causal effect. All tests are performed at 5% significance level. SE = standard error.

|  |  |  |  |  |  |  |
| --- | --- | --- | --- | --- | --- | --- |
|  |  |  |  |  |  |  |
|  |  | Inverse-variance weighted | | MR-Egger regression | | |
|  |  |  |  |  |  |  |
|  | Mean F | Mean estimate | Power to detect | Mean estimate | Power of | Power to detect |
| J | statistic | (mean SE) | causal effect | (mean SE) | MR-Egger test | causal effect |
|  |  |  |  |  |  |  |
| No causal effect: β = 0 | | |  |  |  |  |
|  |  |  |  |  |  |  |
| Scenario (c) – directional pleiotropy, InSIDE satisfied | | |  |  |  |  |
|  |  |  |  |  |  |  |
| 3 | 405.0 | 0.043 (0.028) | 0.139 | 0.002 (0.107) | 0.064 | 0.060 |
| 5 | 296.0 | 0.039 (0.021) | 0.248 | 0.005 (0.059) | 0.079 | 0.056 |
| 10 | 172.0 | 0.039 (0.015) | 0.615 | 0.006 (0.038) | 0.142 | 0.047 |
| 15 | 121.0 | 0.039 (0.012) | 0.803 | 0.006 (0.030) | 0.200 | 0.052 |
| 20 | 93.6 | 0.038 (0.011) | 0.921 | 0.005 (0.025) | 0.259 | 0.048 |
| 30 | 64.4 | 0.038 (0.009) | 0.987 | 0.008 (0.020) | 0.367 | 0.075 |
| 50 | 39.7 | 0.038 (0.007) | 1.000 | 0.010 (0.015) | 0.499 | 0.114 |
| 100 | 20.7 | 0.038 (0.005) | 1.000 | 0.014 (0.010) | 0.717 | 0.295 |
| 150 | 14.2 | 0.038 (0.004) | 1.000 | 0.018 (0.008) | 0.778 | 0.586 |
|  |  |  |  |  |  |  |
|  |  |  |  |  |  |  |

Web Table A2: Performance of inverse-variance weighted and MR-Egger regression estimates in a simulation study for one-sample Mendelian randomization with a null causal effect (β = 0) and a fixed sample size, and varying the number of instruments (J)

### Web Figures

Web Figure A1: Genetic associations with blood pressure and coronary artery disease risk from 29 variants -- scatter plots of minor allele frequency corrected genetic associations with blood pressure ($\hat{\gamma}_{j}^{C}$) against genetic associations with coronary artery disease ($\hat{\Gamma}_{j}$). Left: scatter plot for systolic blood pressure. Right: scatter plot for diastolic blood pressure. The inverse-variance weighted (IVW, red) and MR-Egger (blue) causal effect estimates are also shown as regression slopes.


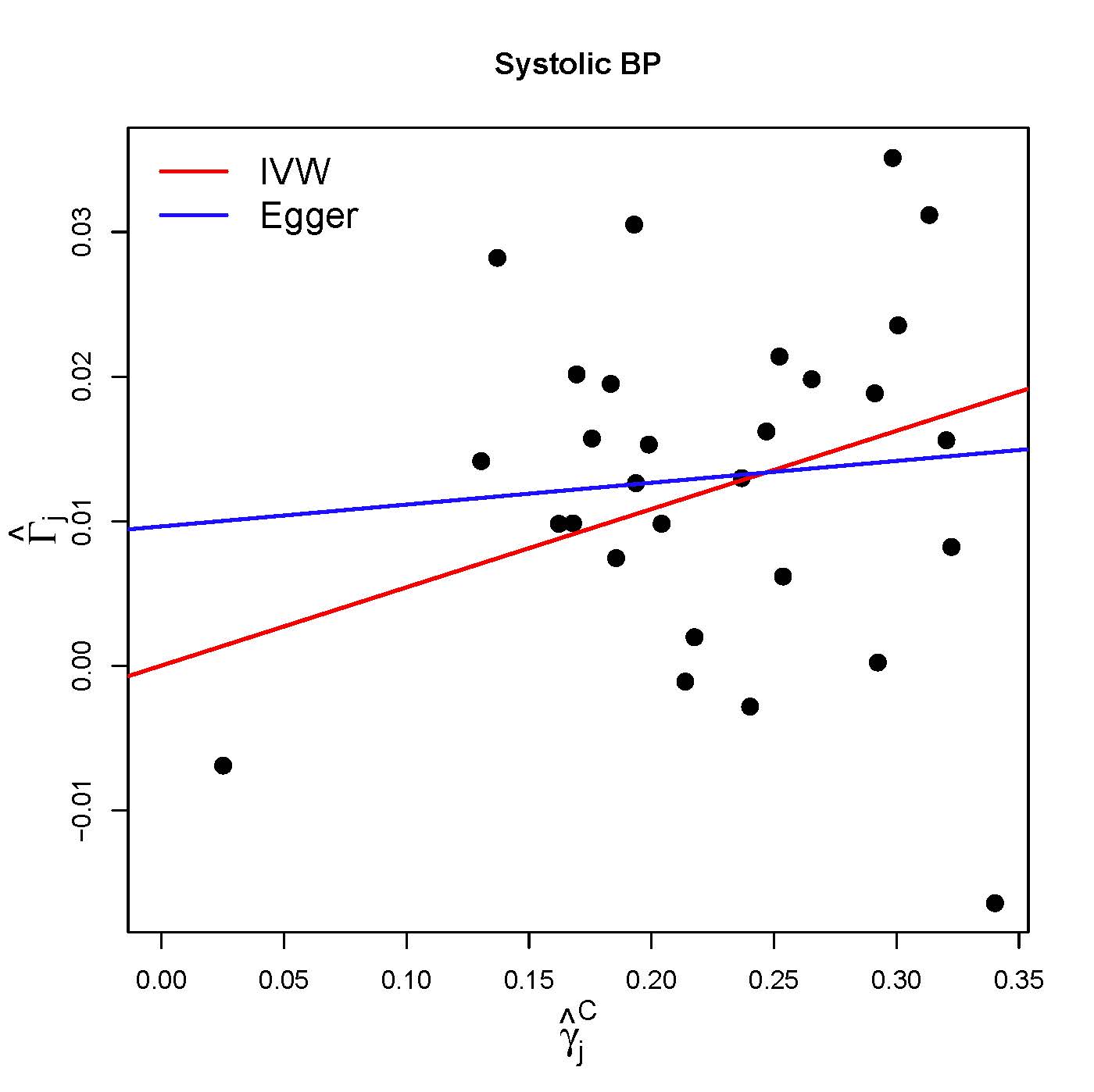

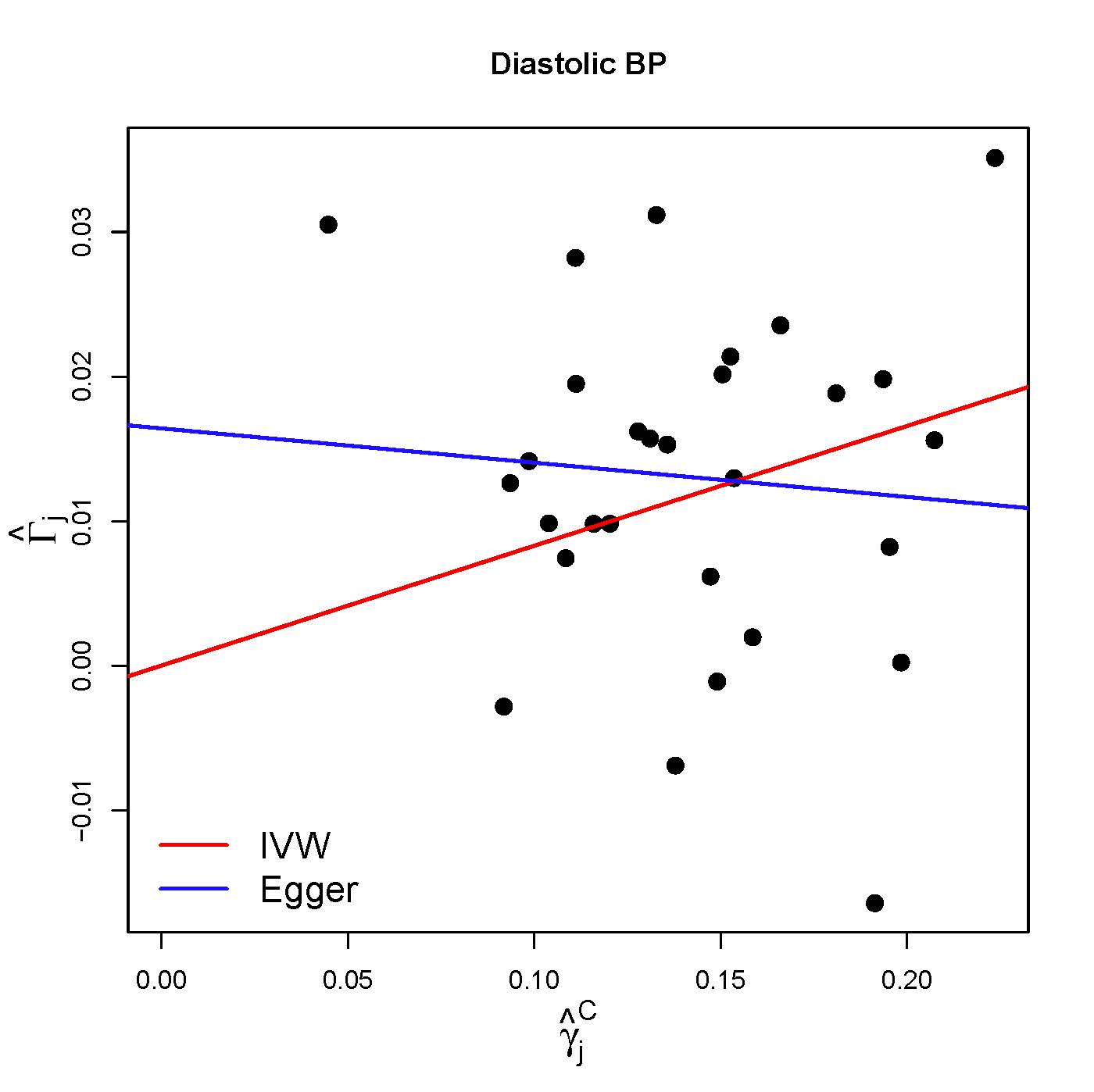


Web Figure A2: Scatter plots of minor allele frequency corrected genetic associations with exposure ($\hat{\gamma}_{j}^{C}$) against genetic associations with the outcome ($\hat{\Gamma}_{j}$) for 50 variants in four scenarios: (a) no pleiotropy; (b) balanced pleiotropy; (c) directional pleiotropy, InSIDE assumption satisfied; and (d) directional pleiotropy, InSIDE assumption not satisfied. The inverse-variance weighted (IVW, red) and MR-Egger (blue) causal effect estimates are also shown.

**
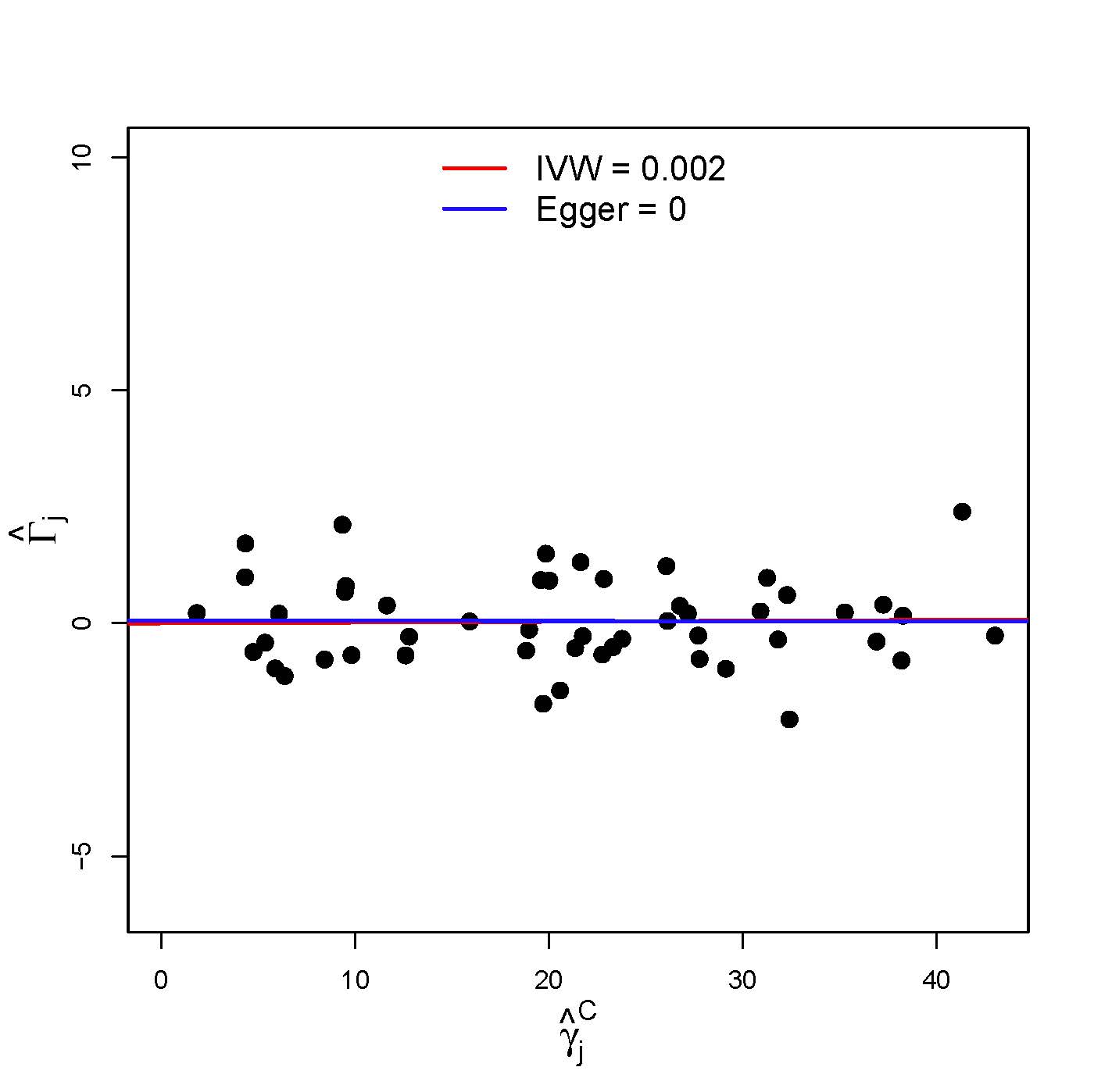

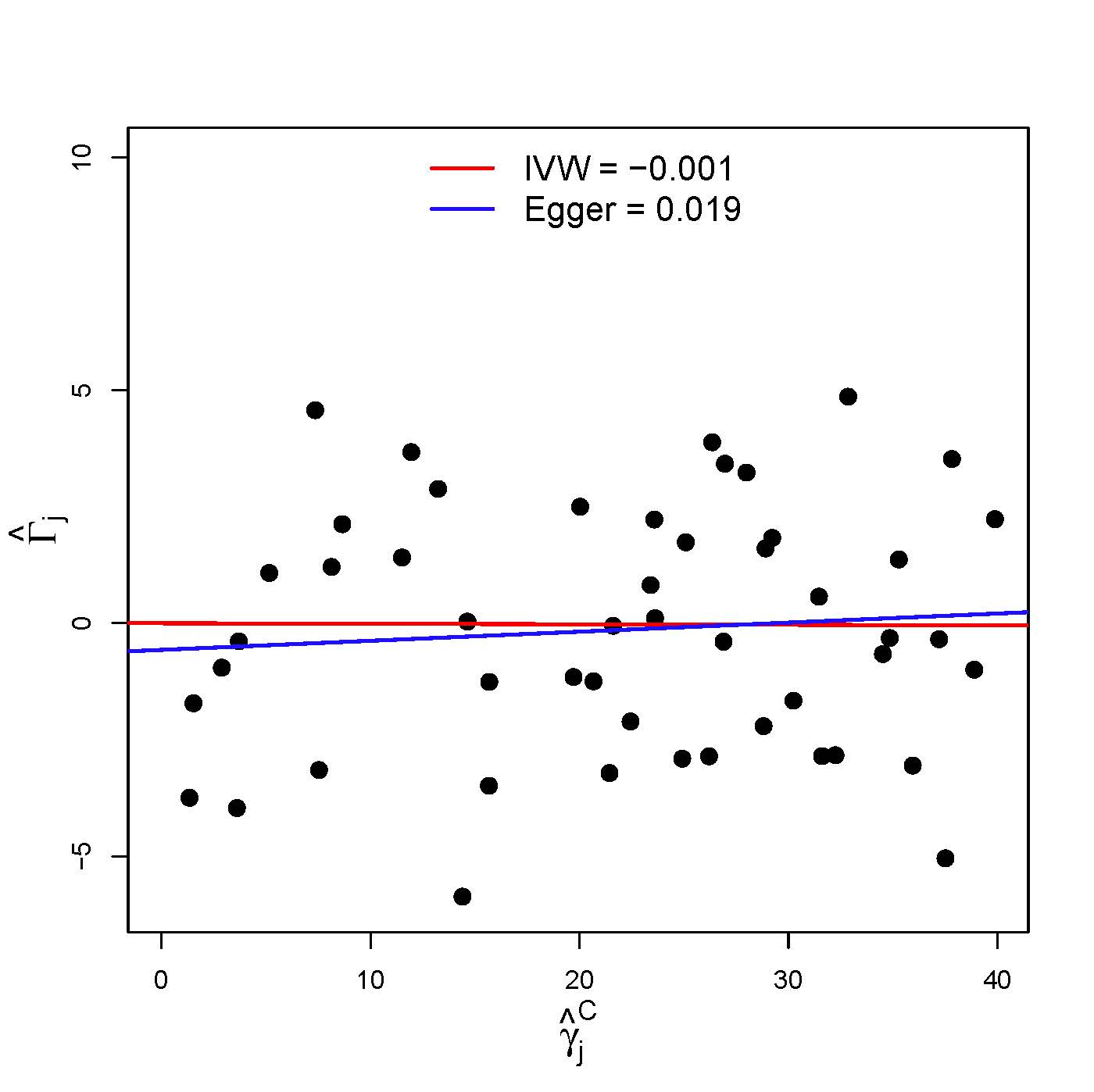
**

**
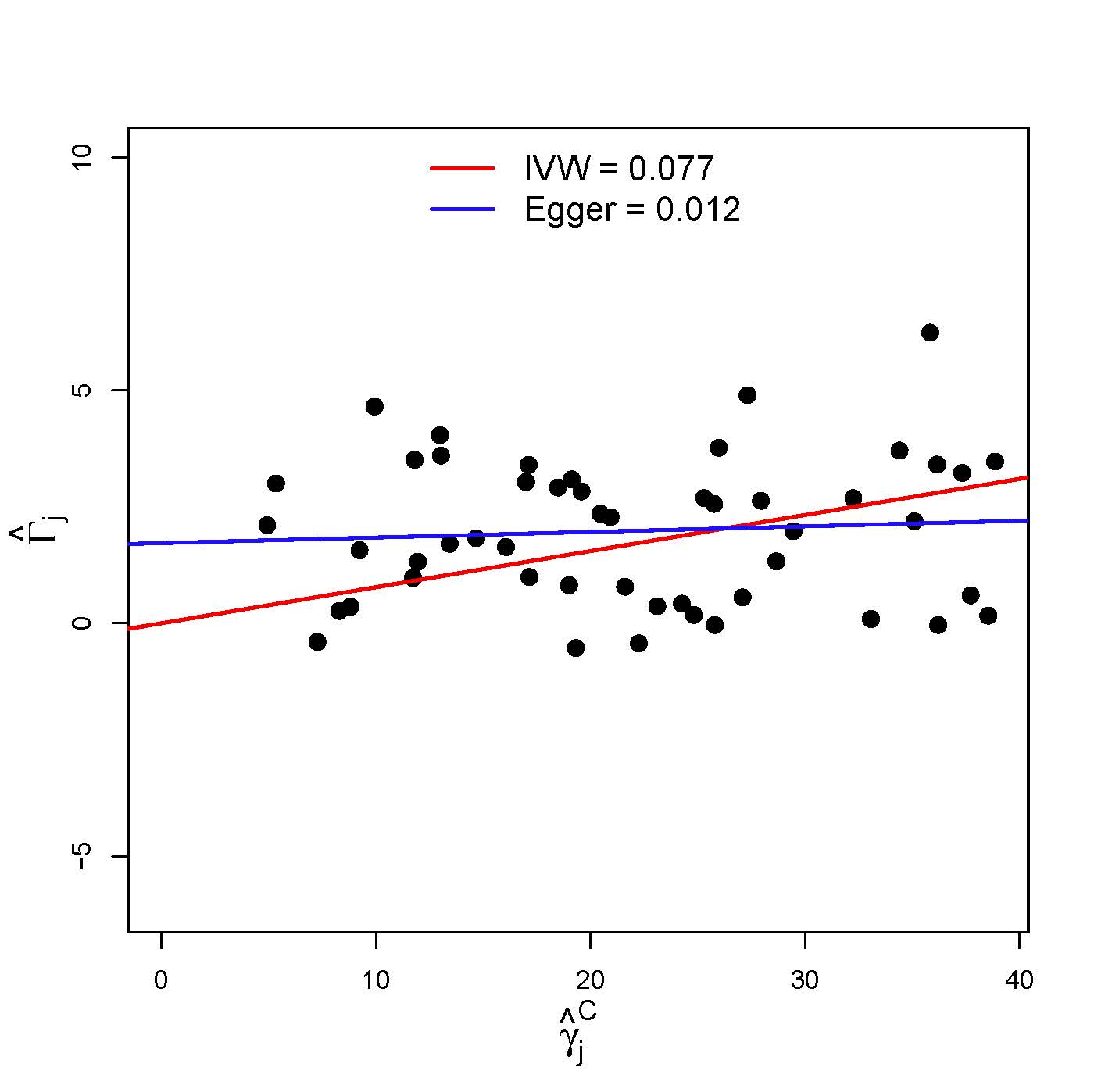

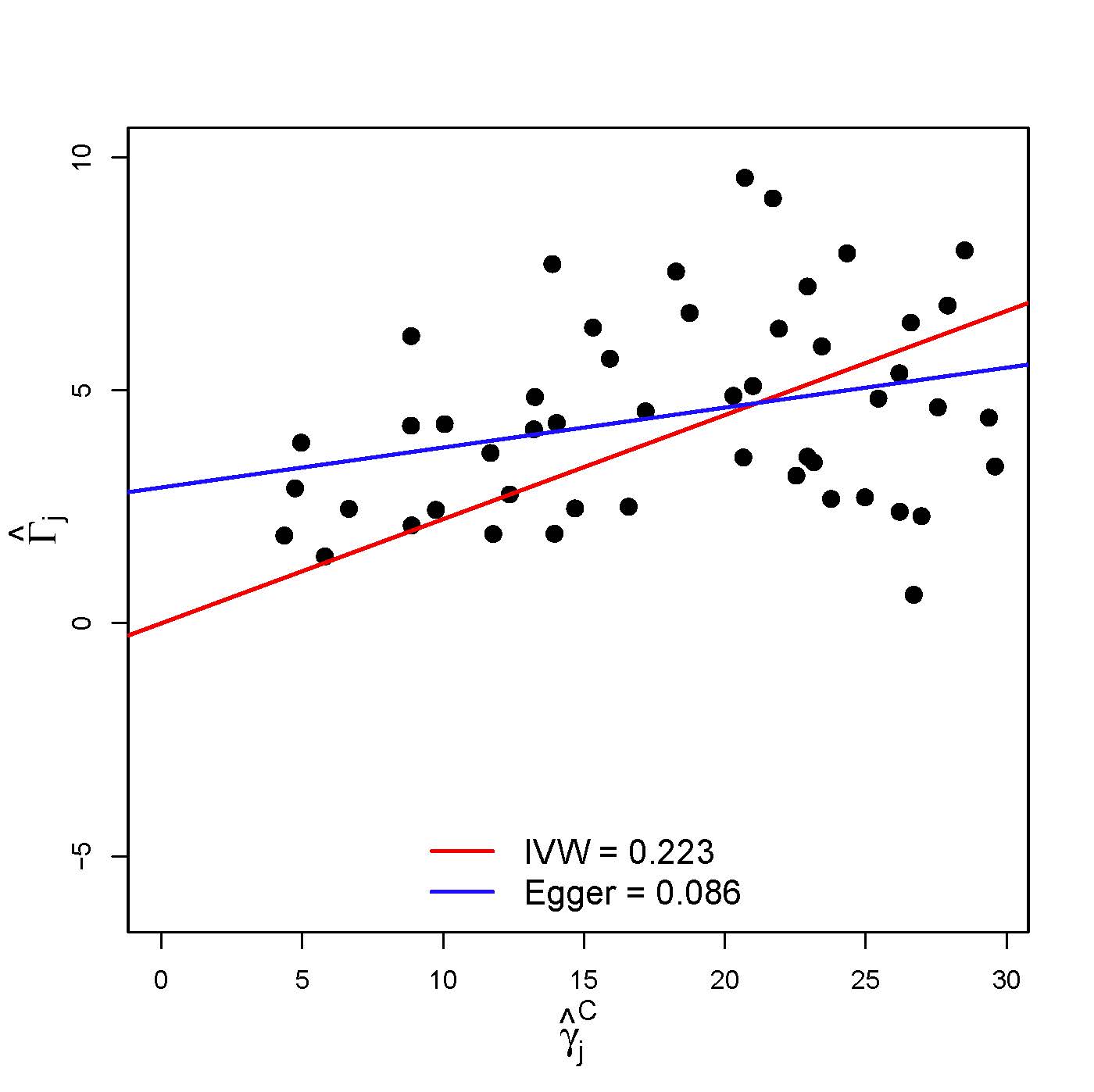
**
